# The effect of Tai Chi Chuan in reducing falls among elderly people: design of a randomized clinical trial in the Netherlands [ISRCTN98840266]

**DOI:** 10.1186/1471-2318-6-6

**Published:** 2006-03-30

**Authors:** Petra EM Zeeuwe, Arianne P Verhagen, Sita MA Bierma-Zeinstra, Erik van Rossum, Marjan J Faber, Bart W Koes

**Affiliations:** 1 Department of General Practice, Erasmus MC, University Medical Center Rotterdam, P.O. Box 1738, 3000 DR Rotterdam, The Netherlands; 2 Department of Health Care Studies, Faculty of Health Sciences, Maastricht University, P.O. Box 616, 6200 MD Maastricht, The Netherlands; 3 Professional University Zuyd, Department of Physiotherapy, P.O. Box 550, 6400 AN Heerlen, The Netherlands; 4 Centre for Quality of Care Research, Radboud University Nijmegen Medical Centre, P.O. Box 1901, 6500 HB, The Netherlands

## Abstract

**Background:**

Falls are a significant public health problem. Thirty to fifty percent of the elderly of 65 years and older fall each year. Falls are the most common type of accident in this age group and can result in fractures and subsequent disabilities, increased fear of falling, social isolation, decreased mobility, and even an increased mortality. Several forms of exercise have been associated with a reduced risk of falling and with a wide range of physiological as well as psychosocial health benefits. Tai Chi Chuan seems to be the most promising form of exercise in the elderly, but the evidence is still controversial.

In this article the design of a randomized clinical trial is presented. The trial evaluates the effect of Tai Chi Chuan on fall prevention and physical and psychological function in older adults.

**Methods/Design:**

270 people of seventy years and older living at home will be identified in the files of the participating general practitioners. People will be asked to participate when meeting the following inclusion criteria: have experienced a fall in the preceding year or suffer from two of the following risk factors: disturbed balance, mobility problems, dizziness, or the use of benzodiazepines or diuretics. People will be randomly allocated to either the Tai Chi Chuan group (13 weeks, twice a week) or the no treatment control group.

The primary outcome measure is the number of new falls, measured with a diary. The secondary outcome measures are balance, fear of falling, blood pressure, heart rate, lung function parameters, physical activity, functional status, quality of life, mental health, use of walking devices, medication, use of health care services, adjustments to the house, severity of fall incidents and subsequent injuries. Process parameters will be measured to evaluate the Tai Chi Chuan intervention. A cost-effectiveness analysis will be carried out alongside the evaluation of the clinical results. Follow-up measurements will be collected at 3, 6 and 12 months after randomization.

**Discussion:**

As far as we know this is the first trial in Europe considering Tai Chi Chuan and fall prevention. This project will answer a pragmatic research question regarding the efficacy of Tai Chi Chuan regarding fall reduction.

## Background

Thirty to fifty percent of the elderly of 65 years and older fall each year [1,2]. About twenty percent of them need medical care after a fall and about six percent of the accidents result in a fracture. Moreover falls can result in disabilities, increased fear of falling, social isolation, decreased mobility and even an increased mortality [3,4]. The risk to fall is strongly related to previous falls, disturbed balance, dizziness, decreased muscular strength, use of benzodiazepines and diuretics and changes in walking pattern [5,6]. Based on these prognostic factors it is feasible to identify elderly who are at risk for falling.

To prevent fall incidents in the elderly exercise programs may be useful. In the most recent Cochrane review [2] on the effectiveness of interventions to prevent fall incidents and in the meta-analysis of the FICSIT (Frailty and Injuries: Cooperative Studies of Intervention Techniques) trials [7] the effectiveness of different exercise programs in healthy elderly populations were examined. In both the review and the meta-analysis exercises seem to have a positive effect to prevent fall incidents, although the evidence is limited. In addition, reviews of Verhagen et al. [8] and Li et al. [9] show that the evidence is still weak for the effect of Tai Chi Chuan in preventing falls, improving balance and improving cardiovascular function.

Tai Chi Chuan is a traditional Chinese exercise that has been practiced for many centuries. It is an integral part of the Traditional Chinese Medicine. It consists of a series of movements (positions) that are performed in a slow and flowing manner. The Tai Chi positions operate on three basic principles. First, the body should be extended and relaxed. Second, the mind must be alert and calm. Last, all body movements require well-coordinated sequencing of segments. The accent is on relaxation and balance control. In the literature many beneficial effects of Tai Chi Chuan are reported, such as improved balance, decreased fall incidents, decreased blood pressure, and increased self-efficacy [9].

Although promising, it is not entirely clear yet whether Tai Chi Chuan can indeed prevent fall incidents in the elderly. Only in one trial it is found to reduce the risk of multiple falls by 47,5% [10]. Since the evidence is still controversial we decided to conduct this study evaluating the effect of Tai Chi Chuan on fall prevention and physical and psychological function in older adults in the Dutch situation.

## Methods/Design

### Study design

**Figure 1:**
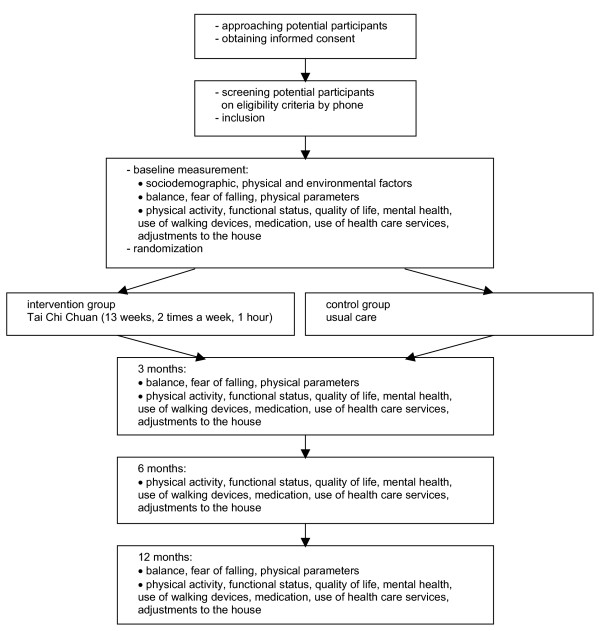
Study design.

This study is a randomized, partially blinded, clinical trial to assess the effectiveness of 'usual care' and Tai Chi Chuan compared to 'usual care' only on fall prevention in the elderly living at home with an increased risk for falling. 'Usual care' means that people can use or apply for all available services in the area. Figure 1 presents the flow diagram of the study design. The Medical Ethics Review Committee at Erasmus MC, University Medical Center Rotterdam, approves the study.

### Study population

General practitioners (GPs) will be informed by mail in Dordrecht and Zwijndrecht (two towns in the west of the Netherlands) about the background and the goal of our research. Using a response fax they can indicate if they are willing to participate or not. If we do not receive a response within a week or after sending a reminder, we will phone them to get clear if they are willing to participate. An appointment will be made with the participating GPs in order to identify eligible people from their patient databases. Eligible are all people of seventy years and older living at home who are at risk for falling. We will select on the basis of in- and exclusion criteria as shown in table 1.

**Table 1:** Inclusion and exclusion criteria

***Inclusion criteria***
- 70 years and older
- living at home
- at least one self-reported fall accident in the preceding year or at least two of the following self-reported risk factors for falling: disturbed balance, mobility problems, dizziness or the use of benzodiazepines or diuretics
***Exclusion criteria***
- progressive disorders:
• neurological disorders (e.g. Parkinson's disease, Alzheimer's disease, multiple sclerosis, epilepsy, grand stroke with rest symptoms, polyneuropathy)
• metastatic/terminal cancer or undergoing a treatment for cancer
- unable to undergo the intervention (e.g. confined to bed, restricted to a wheelchair, unable to walk for eight meters without help, unable to reach the location). These items are stated by the GP after inclusion criteria are checked by phone.
- unable to fill in a Dutch questionnaire
- not available for measurements or intervention or knowing in advance to miss eighty percent of the lessons
- received Tai Chi Chuan in the preceding year

Potential eligible people will receive an information package with detailed information on the study from their GP. Using the included response form they can sign up, ask for additional information or withdraw. In case of withdrawal, people will be kindly requested to write down the reason. In case of willingness to participate they will be asked to sign an informed consent form. A postage free envelope will be included. Reminders will be sent after four weeks.

### Telephone questionnaire

After returning the response form, potential participants will be interviewed by phone. This serves as the screening instrument for final eligibility (see table 1) and partly as baseline measurements. Participants are again informed on the randomization procedures, allowing them another opportunity to reconsider participation before study entry. Potential participants will be asked which study group they prefer to be enrolled in, to evaluate whether patient preferences might influence outcome. Finally, participants will be invited to a location near their homes to conduct the remainder of the baseline measurements.

### Baseline measurements

The baseline measurements consist of the following items. Sociodemographic factors (age, gender, ethnicity, marital status, education, composition of household), physical characteristics (e.g. height, weight, acuity of vision), environmental factors (e.g. pets, adjustments to the house), use of walking devices, medication, and use of health care services (e.g. GP, specialist, physiotherapy, home care/district nurse).

At baseline the following secondary outcome measures are measured: Balance (Berg Balance Scale), fear of falling (The Falls Efficacy Scale), physical parameters (blood pressure and heart rate at rest, FEV1 (i.e. forced expiratory volume during the first second) and PEF (i.e. peak expiratory flow)), physical activity (Physical Activity for the Elderly), functional status (Groningen Activity Restriction Scale), quality of life (EuroQol), and mental health (SF-36). For further details see 'Secondary outcome measures'.

### Sample size and power

The effect of Tai Chi in decreasing fall incidents is conservatively estimated on 25 percent [10]. A power calculation (alpha = 0,05; beta = 0,1; drop out percentage of twenty percent) estimates that 135 participants are needed in each study group. This implies that we intend to enroll 270 participants in total.

### Randomization

After baseline measurements participants will be assigned to one of the four strata based on gender (male/female) and the prognostic factor fall accidents in the preceding year (yes/no). An independent research assistant will perform randomization into either the control or the intervention group using a randomization list generated by a computer. To avoid unequal group sizes a block randomization will be used using block sizes of four. When two persons live together (e.g. married couples) they will be allocated to the same group, i.e. the group to which the first person contacted is allocated to, in order to avoid contamination.

### Masking

GPs will be masked because they are not informed which group the patient is allocated to. Tai Chi Chuan teachers and the participants cannot be masked. Both research assistants who perform the measurements and the researcher who performs the data analyses will be masked.

### Control group

Participants in the control group receive 'usual care' without an additional intervention. 'Usual care' means that they, as before, can use or apply for all available services in the area. Participants in both the control and the intervention group will receive a brochure containing general information on how to prevent falls in and around the house.

### Intervention group

Participants in the intervention group will receive in addition to the 'usual care' Tai Chi Chuan lessons.

The Tai Chi Chuan form is based on the one used by Wolf et al. [11]. This form turned out to be appropriate for the elderly because all components of movement that typically become limited with aging are emphasized. The ten positions are easily comprehensible. It is derived from the Yang style and consists of a form with ten positions: 1. Opening; 2. Grasping the Sparrow's Tail, Left; 3. Grasping the Sparrow's Tail, Right; 4. Cloud Hands; 5. Repulse Monkey; 6. Part Wild Horse's Mane; 7. Brush Knee Twist Step; 8. Lift Kick Left; 9. Lift Kick Right; 10. Closing. The ten positions are linked together in a continuous, smooth-flowing form.

Four Tai Chi Chuan-teachers are available to give the lessons. All are qualified to give the lessons and are experienced in working with elderly. Together they will develop and finalize the Tai Chi Chuan-protocol.

In the literature [8] the recommended frequency of lessons varies from once a day to once a week during a period ranging from 10 weeks to one year. We thought the most optimum and practicable frequency is twice a week for thirteen weeks. Each lesson will last one hour, starting with twenty minutes warming-up and ending with ten minutes cooling down. Hereby Chi Kung exercises will be used. Chi Kung means 'control or mastery of the breath'. Emphasis is placed on relaxation and on contemplation of the breath. In addition to the lessons the participants will be asked to practice the learned Tai Chi Chuan positions at home at least twice a week for about fifteen minutes.

### Outcome measures

The primary outcome measure is new fall accidents. At the end of the baseline measurements participants will receive a fall calendar. They will be asked to fill in daily for a year if they are fallen, nearly fallen or not fallen. A fall is defined as 'an unintentionally coming to rest on the ground, floor, or other lower level'. A near-fall is defined as 'the person seems to fall, but can prevent the fall by catching or leaning on a person or a thing (e.g. a chair, a drawer or a table)' [12].

When a fall or a near-fall is reported people will be asked to give more details (e.g. how it happened, at what time, if they were hurt, if they started to use devices, whether medical care was needed) on an additional standardized form. The forms can be returned in postage free envelopes at the end of each month. If forms are missing or incomplete the research assistant will contact the participant by phone and together they will complete the forms.

### Secondary outcome measures

- Balance is measured with the Berg Balance Scale. It consists of fourteen items that are scored on a five-points ordinal scale [13-15].

- Fear of falling is measured with the Falls Efficacy Scale [16]. It is an oral questionnaire and consists of ten items scored on a four points scale, ranging from 'not concerned at all' to 'very concerned'. Participants are asked about how concerned they are that they might fall while performing a series of ten tasks of progressive difficulty in the home environment.

- Physical parameters: blood pressure and heart rate in rest, FEV1 (i.e. forced expiratory volume during the first second) and PEF (i.e. peak expiratory flow). The latter two are measured by a spirometer.

- Physical activities are measured with the Physical Activity Scale for the Elderly (PASE) [17,18]. It combines the frequency of leisure, household and occupational activities over the previous seven days.

- Functional status is measured with the Groningen Activity Restriction Scale (GARS), covering activities of daily living (ADL) and instrumental activities of daily living (IADL) [19]. It consists of eighteen items scored on a four points scale.

- Quality of life is measured with the EuroQol EQ 5-D. It will be used in the cost-effectiveness analysis [20-22].

- Mental health is measured with the SF-36 (subscales) [23-26].

- Use of walking devices, medication, use of health care services (e.g. GP, specialist, physiotherapy, home care/district nurse), adjustments to the house.

### Follow-up

As shown in table 2 participants will be followed up during twelve months after baseline measurements. At the end of intervention period (i.e. at three month) and twelve months after the baseline measurement all measurements will be performed. At six months only the questionnaire will be send by mail.

**Table 2:** Variables and timing measurements

**Variable**	**Time measured**			
	Baseline	3 months	6 months	12 months

Inclusion and exclusion variables	TQ			
Falls and near-falls	Continuously every day when fall occur			
Severity of fall incidents	Continuously every day when fall occur			
Sociodemografic, physical and environmental factors	WQ			
Balance (Berg Balance Scale)	M	M		M
Fear of falling (Falls Efficacy Scale)	OQ	OQ		OQ
Physical parameters (blood pressure, heart rate, lung function parameters)	M	M		M
Physical activity (Physical Activity Scale for the Elderly)	WQ	WQ	PQ	WQ
Functional status (Groningen Activity Restriction Scale)	WQ	WQ	PQ	WQ
Quality of life (EuroQol)	WQ	WQ	PQ	WQ
Mental health (SF-36)	WQ	WQ	PQ	WQ
Use of walking devices, medication, use of health care services, adjustments to the house	WQ	WQ	PQ	WQ
Compliance to intervention		X		

TQ = telephone questionnaire; WQ = written questionnaire; OQ = oral questionnaire; PQ = written questionnaire send by post; D = diary, by self report, concurrent registration; M = measurement (e.g. by a test); X = reported by teacher

Questionnaires will be checked on missing data and completed with the participants by phone.

In the invitation letter to visit the location for the follow-up measurement at three and twelve months, participants will be asked not to tell the research assistant whether they are allocated to the intervention or the control group. Moreover a reminder sign will be placed on the location. In this way the research assistant will remain masked.

Every effort will be made to keep the loss to follow-up as small as possible. When the questionnaire sent after six months is not received within two weeks, a phone call is made in order to remind the participant.

### Process evaluation

At the end of the intervention period participants and teachers will be asked to fill out a questionnaire to evaluate the Tai Chi Chuan course. This includes the appreciation of the course, the expected benefit, et cetera. The teachers will report their experiences with the protocol, and fill out an attendance list per lesson. Reasons for noncompliance will be written down.

Dropouts will be phoned to get information on the reason of stopping with the course.

These results can be used to optimize the content and implementation of the courses in future. Moreover, it can provide information explaining measured effects or explaining why expected effects were not identified. Moreover a comparison between these self-reported values and the measured values (i.e. Berg Balance Scale) can be made.

### Data entry

Data will be entered in SPSS using a protocol. There will be a check on a random sample of ten percent of the data. Thereafter an overall check will be done using frequency tables to detect unusual values, inconsistencies and missing values.

### Statistical analyses

To assess if randomization was successful, descriptive statistics of baseline characteristics will be used. In case clinically relevant baseline differences regarding prognosis of future incidence of falls is present, effect estimates will be adjusted using multivariate analysis.

Dropouts are defined as participants who stop participating during the first three months (i.e. intervention period). Non-compliers are defined as participants who miss more than twenty percent of the lessons.

The analysis will be performed using the intention-to-treat principle, e.g. all patients are included as randomized irrespective of compliance. Missing values are assigned the last available valid score.

We will evaluate the effect of the intervention in reducing the number of fall incidents using the Student's t-test and survival techniques. To analyze the secondary outcome measures the Student's t-test, the Mann-Whitney test or the Chi-square test will be used according to the type of variable.

When drop out is higher than fifteen percent and/or the compliance is lower than seventy percent an additional per-protocol analysis will be carried out. These are restricted to those participants who complied fully with the intervention protocol and outcome measures.

At the end of the analyses the allocation of participants will be disclosed.

A cost-effectiveness analysis will be conducted alongside the analysis of the clinical data.

Direct costs (such as medication, additional therapy, visits to health care providers) as well as indirect costs (such as disability) measured by the EuroQol, will be related to the primary outcome measure (fall incidents).

### Time plan

The recruitment of the GPs, followed by the search in the patient databases started in September 2003. Thereafter the participants were invited, screened and included. The first intervention group started in February 2004. Follow up will continue during 2006.

## Discussion

As far as we know this is the first trial in Europe considering Tai Chi Chuan and fall prevention. In the United States some similar trials are done, but circumstances differ from Europe since elderly often live in communities. We have to recruit participants via their GP.

Most trials on the effect of Tai Chi Chuan are focused on intermediate outcome measures such as balance, muscular strength, whereas we measure fall incidents.

In this study we use the highest methodological standards. Masking is performed when possible. Moreover randomization will be checked to make clear if baseline characteristics in both groups are equal. If needed adjustments will be made.

The intervention and control group not only differ regarding the intervention, but also in the amount of attention the individuals receive. As social relations integral to the exercise environment are determinants of subjective well-being in older adults [27] we considered to make a third group of elderly, receiving another group-based activity to assess the positive effect of social function of the group. But as this project was restricted in time, the funding organization and we decided to work with two groups. This means that this project will answer a pragmatic research question regarding the efficacy of Tai Chi Chuan regarding fall reduction.

To keep the drop out as low as possible the Tai Chi Chuan-lessons are given at a location close to where the elderly live, the travel costs of the participants will be refunded and as needed we organize transportation.

## Competing interests

The author(s) declare that they have no competing interests.

## Authors' contributions

APV and BWK developed the design of the randomized clinical trial and are co-author. PZ completed the study design and will perform the research. All authors have read and approved the final manuscript. All results out of this trial will be published using the International Standardised Randomised Controlled Trial Number (ISRCTN).

## Pre-publication history

The pre-publication history for this paper can be accessed here:


